# Investigations of anticholinestrase and antioxidant potentials of methanolic extract, subsequent fractions, crude saponins and flavonoids isolated from *Isodon rugosus*

**DOI:** 10.1186/0717-6287-47-76

**Published:** 2014-12-26

**Authors:** Anwar Zeb, Abdul Sadiq, Farhat Ullah, Sajjad Ahmad, Muhammad Ayaz

**Affiliations:** Department of Pharmacy, University of Malakand, Chakdara, Dir Pakistan

**Keywords:** Anticholinesterase, Antioxidant, Flavonoids, Phenolics, Saponins, *Isodon rugosus*

## Abstract

**Background:**

Based on the ethnomedicinal uses and the effective outcomes of natural products in various diseases, this study was designed to evaluate *Isodon rugosus* as possible remedy in oxidative stress, alzheimer’s and other neurodegenerative diseases. Acetylecholinestrase (AChE) and butyrylcholinesterase (BChE) inhibitory activities of crude methanolic extract (Ir.Cr), resultant fractions (*n*-hexane (Ir.Hex), chloroform (Ir.Cf), ethyl acetate (Ir.EtAc), aqueous (Ir.Aq)), flavonoids (Ir.Flv) and crude saponins (Ir.Sp) of *I. rugosus* were investigated using Ellman’s spectrophotometric method. Antioxidant potential of *I. rugosus* was determined using DPPH, H_2_O_2_ and ABTS free radicals scavenging assays. Total phenolic and flavonoids contents of plant extracts were determined and expressed in mg GAE/g dry weight and mg RTE/g of dry sample respectively.

**Results:**

Among different fractions Ir.Flv and Ir.Cf exhibited highest inhibitory activity against AChE (87.44 ± 0.51, 83.73 ± 0.64%) and BChE (82.53 ± 0.71, 88.55 ± 0.77%) enzymes at 1 mg/ml with IC_50_ values of 45, 50 for AChE and 40, 70 μg/ml for BChE respectively. Activity of these fractions were comparable to galanthamine causing 96.00 ± 0.30 and 88.61 ± 0.43% inhibition of AChE and BChE at 1 mg/ml concentration with IC_50_ values of 20 and 47 μg/ml respectively. In antioxidant assays, Ir.Flv, Ir.Cf, and Ir.EtAc demonstrated highest radicals scavenging activities in DPPH and H_2_O_2_ assays which were comparable to ascorbic acid. Ir.Flv was found most potent with IC_50_ of 19 and 24 μg/ml against DPPH and H_2_O_2_ radicals respectively. Whereas antioxidant activates of plant samples against ABTS free radicals was moderate. Ir.Cf, Ir.EtAc and Ir.Cr showed high phenolic and flavonoid contents and concentrations of these compounds in different fractions correlated well to their antioxidant and anticholinestrase activities.

**Conclusion:**

It may be inferred from the current investigations that the Ir.Sp, Ir.Flv and various fractions of *I. rugosus* are good sources of anticholinesterase and antioxidant compounds. Different fractions can be subjected to activity guided isolation of bioactive compounds effective in neurological disorders.

## Background

Alzheimer's disease (AD) is a chronic neurodegenerative disorder effecting more than twenty millions people globally and is the most common cause of dementia in elder population [1, 2]. AD is characterized by degeneration of neurological function, presence of extra-neuronal amyloid deposits, neuritic plaques, gradual deficits in various neurotransmitters leading to decline in the levels of acetylcholine (ACh) and loss of cognitive abilities [1]. Inhibitors of acetylecholinestrase (AChE) and butyrylcholinestrase (BChE), key enzymes involved in the degradation of neurotransmitter ACh, have been shown to function by restoring the level of ACh in the synaptic region and thus reinstate deficient cholinergic neurotransmission [3, 4]. Since the discovery of cholinergic deficits in patients suffering from neurological disorders, inhibition of these enzymes is the main target in the treatment of AD, senile dementia, Parkinsonism, ataxia and myasthenia gravis [5, 6]. Synthetic drugs used in the treatment of cognitive dysfunction associated with AD and other diseases include tacrine, donepezil and rivastigmine [7]. But these drugs are associated with adverse effects including gastrointestinal disturbances, hepatotoxicity and bioavailability problems [8, 9], which necessitates the development of better AChE and BChE inhibitors from natural resources i.e. galantamine.

Reactive oxygen species (ROS) are produced during aerobic respiration and other redox processes in the body. These ROS can attack some important biomolecules like enzymes, lipids, proteins, DNA and RNA leading to cellular damage and plays a major role in aging process [10]. To counteract oxidative stress, human body has several defense mechanisms including antioxidant enzymes and non-enzymatic compounds. But excess of free radicals make the organism unable to scavenge all ROS. These ROS are implicated in the development of some chronic diseases including cardiovascular diseases, cancer, diabetes, nephritis, rheumatism and aging. Oxidative stress has been reported to play a key role in the progression of neurodegenerative diseases like AD and Parkinson's disease [11, 12]. Generally consumption of herbs, fruits and vegetables are beneficial for health due to the presence of protective antioxidant phyto-nutrients present in them [13, 14]. Synthetic antioxidant compounds including gallic acid esters, butylated hydroxy toluene (BHT), tertiary butylated hydroquinone and butylated hydroxy anisole (BHA) are associated with severe adverse effects and hence their use is limited. Consequently, there is need to discover new antioxidants from natural sources [15]. In this regard antioxidant potential of numerous plants has been reported [16–19]. In antioxidant compounds, flavonoids and phenolic compounds are very important. These compounds has the ability to scavenge free radicals effectively due to the presence of hydroxyl groups and conjugated system [20].

*I. rugosus* belongs to *Labiateae*, a family rich in species containing huge number of pharmacologically active compounds. The *Isodon* species are traditionally used as anticancer, antimicrobial, insecticidal, antioxidants and as anthelmintic [21–24]. *I. rugosus* is used traditionally in the management of hypertension, rheumatism, tooth-ache, and pyrexia [25]. Ethno-medicinally, this is used in the treatment of skin diseases, ear, nose and throat infections and the treatment of intestinal disorders are also reported [26]. In an effort to discover new sources which can potentially be used in the treatment of oxidative stress, AD and other neurological disorders, *I. rugosus* was investigated for anticholinesterase and antioxidant potentials.

## Results

### Total phenolic and flavonoid contents

Results of total phenolic and flavonoids contents in different fractions of *I. rugosus* are summarized in Table 1. Results indicate that Ir.Cf, Ir.EtAc and Ir.Cr exhibited high phenolic contents, i.e. 82.60 ± 0.68, 67.45 ± 0.83 and 65.51 ± 1.67 mg GAE/g of dry sample respectively. Whereas, Ir.Cf, Ir.Cr and Ir.EtAc showed highest flavonoids content i.e. 91.45 ± 1.12, 71.73 ± 0.69 and 68.46 ± 1.83 mg RTE/g of sample respectively. Phenolic compounds show greater antioxidant activity due to the presence of hydroxyl groups and conjugated system in their chemical strucutre [27, 28]. Phenolic and flavonoid contents in different fractions of plant correlated well to the antioxidant activity.

**Table 1:** **Total phenolics and total flavonoids of Ir.Cr and subfractions of** ***I*** **.** ***rugosus***

Samples	Total phenolics (mg GAE/g of sample)	Total flavonoids (mg RTE/g of sample)
**Ir.Cr**	65.51 ± 1.67	71.73 ± 0.69
**Ir.Hex**	41.38 ± 1.15	44.40 ± 2.76
**Ir.Cf**	82.60 ± 0.68	91.45 ± 1.12
**Ir.EtAc**	67.45 ± 0.83	68.46 ± 1.83
**Ir.Aq**	18.11 ± 2.25	27.12 ± 1.33

Each value in the table is represented as mean ± SEM (N = 3).

*Abbreviations: Ir.Cr* Crude methanolic extract, *Ir.Hex* *n*-hexane fraction, *Ir.Cf* Chloroform fraction, *Ir.EtAc* Ethyl acetate fraction, *Ir.Aq* Aqueous fraction.

### Anticholinesterase assays

Results of AChE and BChE inhibitory potentials of *I. rugosus* at various tested concentrations are summarized in Table 2. In AChE inhibition assay, Ir.Flv, Ir.Cf and Ir.Sp showed highest activity against AChE causing 87.44 ± 0.51, 83.73 ± 0.64 and 77.85 ± 0.56% inhibitions at 1 mg/ml respectively. For these fractions IC_50_ values were 45, 50 and 75 μg/ml respectively. Standard drug galanthamine inhibited AChE enzyme by 96.00 ± 0.30% at 1 mg/ml concentration with IC_50_ value of 20 μg/ml. Percent inhibitions of these fractions were comparable to the standard drug. AChE inhibition activity of all other fractions was less than 60%. On the other hand, Ir.Cf, Ir.Flv and Ir.EtAc were most effective fractions against BChE enzyme, causing 88.55 ± 0.77, 82.53 ± 0.71 and 76.37 ± 0.68% inhibitions at 1 mg/ml concentration respectively. Ir.Cf, Ir.Flv and Ir.EtAc were most potent, presenting IC_50_ of 70, 40 and 52 μg/ml respectively. BChE inhibitory activities of these fractions were comparable to galanthamine result with percent inhibition of 88.61 ± 0.43% at 1 mg/ml with IC_50_ value of 47 μg/ml.

**Table 2:** **Percent AChE and BChE inhibition potentials of** ***I. rugosus*** **at various tested concentrations**

Samples	Concentrations (μg/ml)	Percent AChE (mean ± SEM)	AChE	Percent	BChE
			IC _50_	BChE	IC _50_
			(μg/ml)	(mean ± SEM)	(μg/ml)
Ir.Sp	1000	77.85 ± 0.56***	75	69.48 ± 0.74***	210
	500	71.64 ± 0.75***		62.62 ± 0.40***	
	250	62.58 ± 0.77***		51.60 ± 0.46***	
Ir.Cr	1000	71.75 ± 0.63***	140	68.68 ± 0.49***	145
	500	63.58 ± 0.70***		61.72 ± 0.66***	
	250	56.61 ± 0.53***		55.46 ± 0.63***	
Ir.Hex	1000	64.79 ± 0.62***	630	62.61 ± 0.77***	380
	500	45.45 ± 0.49***		54.60 ± 0.80***	
	250	43.75 ± 0.58***		43.83 ± 0.56***	
Ir.Cf	1000	83.73 ± 0.64***	50	88.55 ± 0.77^ns^	70
	500	79.47 ± 0.56***		83.65 ± 0.77^ns^	
	250	67.81 ± 0.60***		67.58 ± 0.74***	
Ir.EtAc	1000	71.74 ± 0.61***	170	76.37 ± 0.68***	52
	500	69.68 ± 0.60***		72.66 ± 0.78***	
	250	52.63 ± 0.76***		63.62 ± 0.74***	
Ir.Aq	1000	66.79 ± 0.63***	340	71.62 ± 0.74***	305
	500	59.67 ± 0.61***		63.86 ± 0.60***	
	250	41.69 ± 0.77***		44.48 ± 0.64***	
Ir.Flv	1000	87.44 ± 0.51***	45	82.53 ± 0.71***	40
	500	73.76 ± 0.58***		76.52 ± 0.68***	
	250	71.54 ± 0.50***		68.51 ± 0.77***	
Galanthamine	1000	96.00 ± 0.30	20	88.61 ± 0.43	47
	500	91.26 ± 1.27		83.25 ± 1.40	
	250	82.91 ± 1.30		74.03 ± 0.86	

The data is represented as mean ± SEM, (N = 3). Values significantly different as compare to positive control, *P < 0.05, **P < 0.01, ***P < 0.001.

*Abbreviations: Ir.Cr* Crude methanolic extract, *Ir.Hex* *n*-hexane fraction, *Ir.Cf* Chloroform fraction, *Ir.EtAc* Ethyl acetate fraction, *Ir.Aq* Aqueous fraction, *Ir.Flv* Crude flavonoids, *Ir.Sp* Crude Saponins.

### Antioxidant assays

#### DPPH free radicals scavenging effect

**Figure 1:**
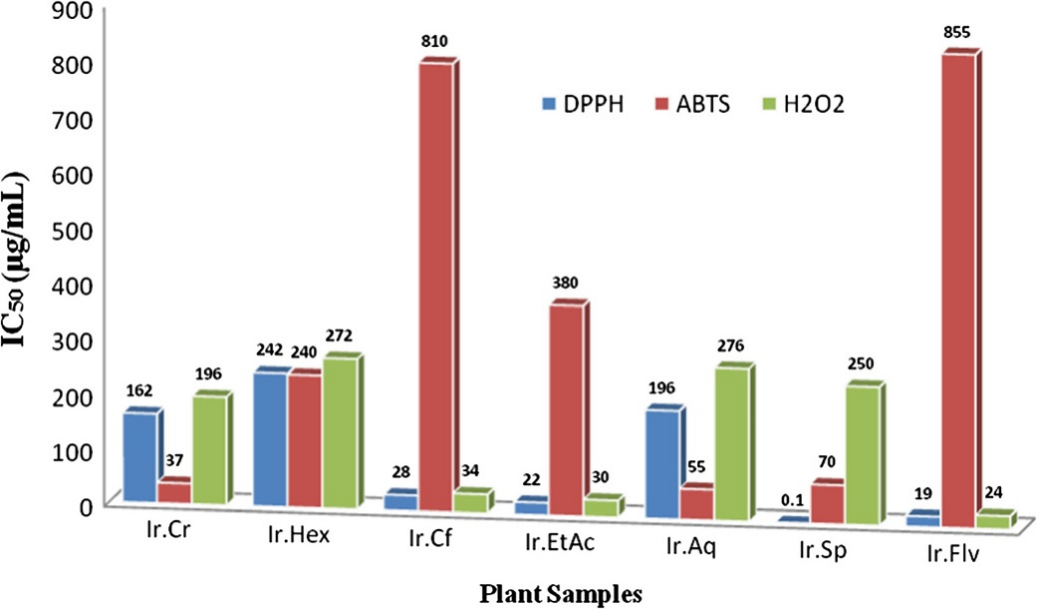
**IC50 values of various samples against DPPH, H2O2 and ABTS free radicals.** Abbreviations: Ir.Cr: Crude methanolic extract; Ir.Hex: *n*-hexane fraction; Ir.Cf: Chloroform fraction; Ir.EtAc: Ethyl acetate fraction; Ir.Aq: Aqueous fraction; Ir.Flv: Crude flavonoids; Ir.Sp: Crude Saponins.

**Figure 2:**
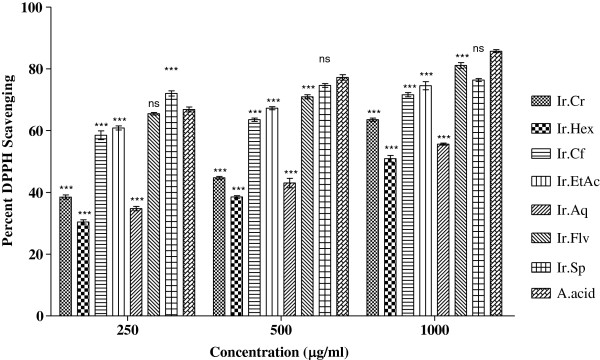
**Antioxidant potential of plant extracts using DPPH assay.** Values represent % radical scavenging (mean ± SEM) of three replicates. Values significantly different as compare to positive control *P < 0.05, **P < 0.01, ***P < 0.001. Abbreviations: Ir.Cr: Crude methanolic extract; Ir.Hex: *n*-hexane fraction; Ir.Cf: Chloroform fraction; Ir.EtAc: Ethyl acetate fraction; Ir.Aq: Aqueous fraction; Ir.Flv: Crude flavonoids; Ir.Sp: Crude Saponins; A.acid: Ascorbic acid.

Analysis of plant samples against DPPH free radicals revealed that Ir.Flv, Ir.Sp, Ir.EtAc and Ir.Cf were most effective causing 81.1 ± 0.90, 76.36 ± 0.48, 74.53 ± 1.34 and 71.63 ± 0.67% scavenging respectively at 1 mg/ml concentration. Median inhibitory concentrations (IC_50_) were 19, < 0.1, 22 and 28 μg/ml for Ir.Flv, Ir.Sp, Ir.EtAc and Ir.Cf respectively as shown in Figure 1. Ascorbic acid (Positive control) cause 85.71 ± 0.49% inhibition of DPPH radicals at 1 mg/ml concentration and IC_50_ was 52 μg/ml. Potency wise activity of Ir.Flv, Ir.Sp, Ir.EtAc and Ir.Cf was better than ascorbic acid. Other fractions were effective in concentration dependent manner. DPPH scavenging activity for all the tested samples were in ascending order of Ir.Flv > Ir.EtAc > Ir.Cf > Ir.Cr > Ir.Aq > Ir.Hex, shown in Figure 2.

#### Hydrogen peroxide free radicals scavenging effect

**Figure 3:**
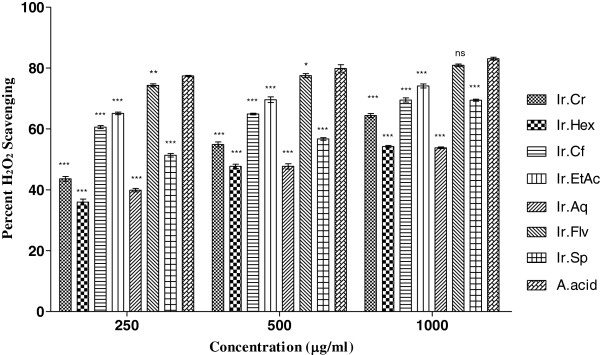
**Antioxidant potential of plant extracts using H**_**2**_ **O**_**2**_ **assay.** Values represent % radical scavenging (mean ± SEM) of three replicates. Values significantly different as compare to positive control, *P < 0.05, **P < 0.01, ***P < 0.001. Abbreviations: Ir.Cr: Crude methanolic extract; Ir.Hex: *n*-hexane fraction; Ir.Cf: Chloroform fraction; Ir.EtAc: Ethyl acetate fraction; Ir.Aq: Aqueous fraction; Ir.Flv: Crude flavonoids; Ir.Sp: Crude Saponins; A.acid: Ascorbic acid.

In H_2_O_2_ free radicals scavenging activity, Ir.Flv, Ir.EtAc, Ir.Sp, Ir.Cf and Ir.Cr showed highest activities causing 78.9 ± 0.41, 74.1 ± 0.67, 69.5 ± 0.67, 69.50 ± 0.67 and 64.4 ± 0.64% inhibition at 1 mg/ml concentration (Figure 3). For these fractions IC_50_ were 24, 30, 250, 34 and 196 μg/ml respectively, as shown in Figure 1. Ascorbic acid inhibition was 83.08 ± 0.47% at the same tested concentration with IC_50_ 24 μg/ml.

#### ABTS free radicals scavenging effect

**Figure 4:**
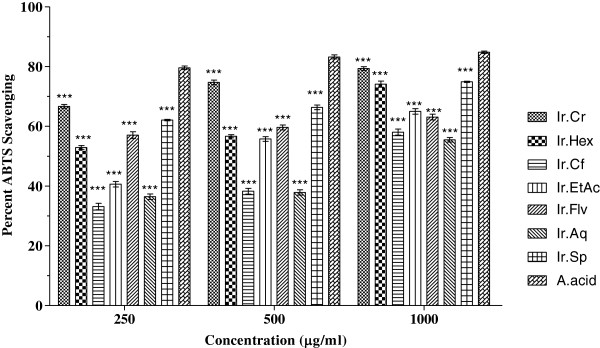
**Antioxidant potential of plant extracts using ABTS assay.** Values represent % radical scavenging (mean ± SEM) of three replicates. Values significantly different as compare to positive control, *P < 0.05, **P < 0.01, ***P < 0.001. Abbreviations: Ir.Cr: Crude methanolic extract; Ir.Hex: *n*-hexane fraction; Ir.Cf: Chloroform fraction; Ir.EtAc: Ethyl acetate fraction; Ir.Aq: Aqueous fraction; Ir.Flv: Crude flavonoids; Ir.Sp: Crude Saponins; A.acid: Ascorbic acid.

In ABTS free radicals scavenging assay, Ir.Cr, Ir.Sp, Ir.Hex, Ir.EtAc and Ir.Flv showed highest activity causing 79.35 ± 0.61, 74.89 ± 0.22, 74.1 ± 1.04, 64.99 ± 0.92 and 57.04 ± 1.12% scavenging respectively at 1 mg/ml concentration (Figure 4). For these fractions, the IC_50_ were 37, 70, 240 and 380 μg/ml respectively, as shown in Figure 1. The ascorbic acid inhibition was 84.78 ± 0.40% at 1 mg/ml with IC_50_ of 50 μg/ml.

### Correlation of total phenolic contents and various activities

**Figure 5:**
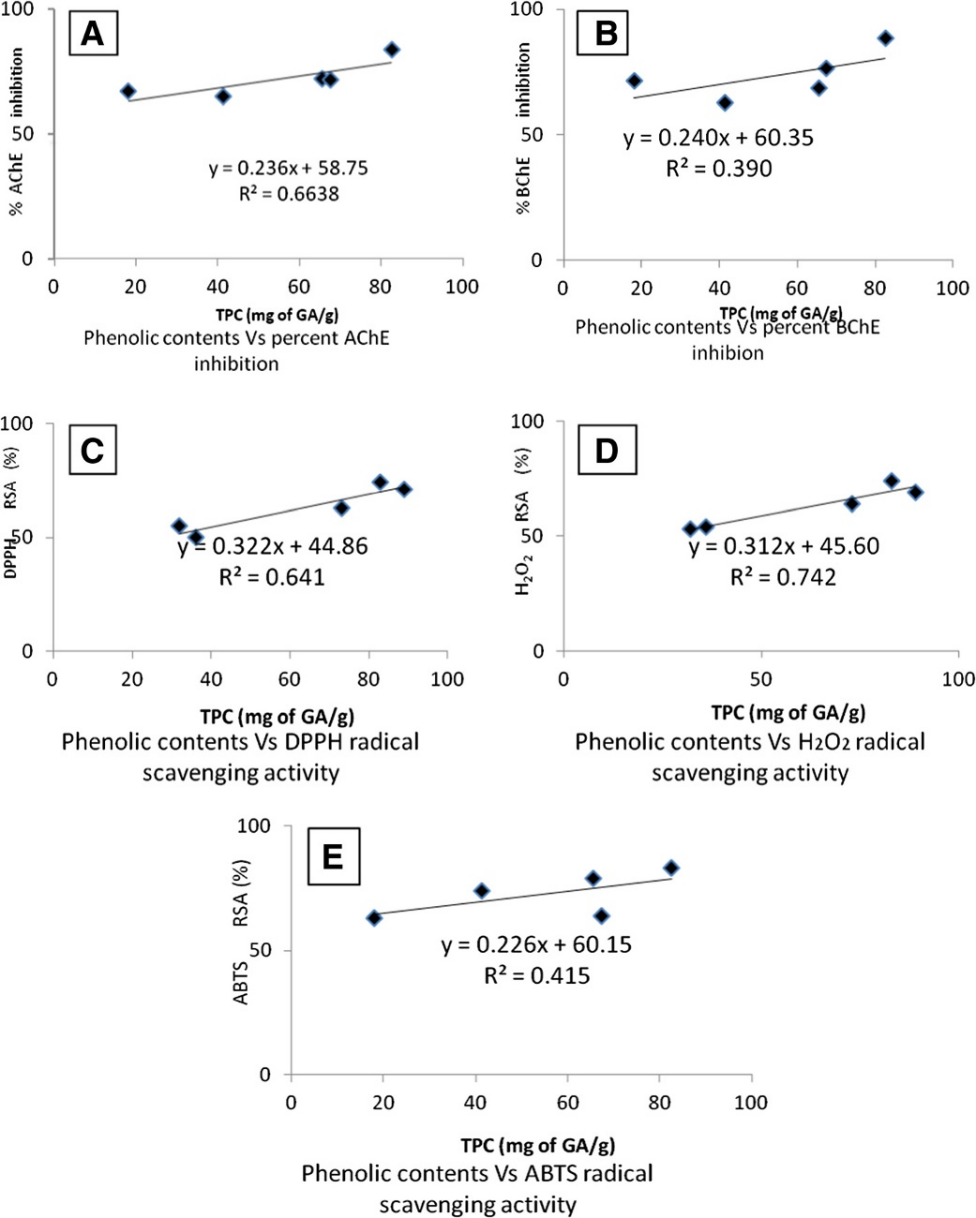
**Linear correlation for total phenolics Vs AChE (A), BChE (B), DPPH (C), H**_**2**_ **O**_**2**_ **(D) and ABTS (E) activities.**

Regression and correlation of total phenolic contents and various assays have been summarized in Figure 5 (A, B, C, D and E). The correlation coefficient (R^2^ = 0.742) obtained by plotting H_2_O_2_ scavenging assay versus total phenolic contents was the most prominent one. The regression line for H_2_O_2_ (y = 0.312x + 45.60) went parallel with the total phenolic contents. Similarly, the correlation co-efficients of ABTS and DPPH were calculated as 0.415 and 0.641 respectively. Furthermore, the regression line obtained for AChE (y = 0.236x + 58.75) and BChE (y = 0.240x + 60.35) goes moderately parallel with the total phenolic contents having correlation coefficient 0.663 and 0.390 respectively.

## Discussion

Antioxidant compounds are useful neuroprotective agents in the therapy of early stages of Alzheimer’s disease (AD) [29]. Though exact origin of AD is unknown, the diminution of neurotransmitters like ACh caused by the enzymes AChE and BChE are clearly involved in the etiology of this disease [30]. One treatment strategy is to enhance cholinergic functions by the use of AChE and BChE inhibitors to augment the amount of ACh present in the synapses between cholinergic neurons. In this regard, use of selective inhibitors for AChE and BChE has attracted particular attention in the treatment of the Alzheimer-type dementia [31]. In recent times, BChE received particular attention since it is a co-regulator of cholinergic neurotransmission and its pharmacological activity is increased in AD and associated with all neuropathological lesions in this disorder [32]. Even though selective inhibitors of BChE from synthetic origin, based on quinazolinimines and lipoic acid have been reported [33]. The search for more efficient and less expensive natural substances is a mandatory research branch [29]. Use of AChE inhibitors like physostigmine or tacrine is restricted due to limitations such as short half life and serious side effects like hepatotoxicity. Alkylpyridium polymers, dehydroevodiamine (DHED) and carbamate type AChE inhibitors have been recently reported, but due to bioavailability problems and side effects, there is still great interest in searching better AChE inhibitors from natural sources [34].

Results of the current study revealed that Ir.Flv, Ir.Cf and Ir.Sp possesses highest inhibitory potential against AChE causing 87.44 ± 0.51, 83.73 ± 0.64 and 77.85 ± 0.56% enzyme inhibitions respectively at 1 mg/ml concentration. These fractions were most potent among others with IC_50_ values of 45, 50 and 75 μg/ml. AChE inhibitory activities of these fractions were comparable to standard drug galanthamine, whose percent inhibition was 96.00 ± 0.30 at the same tested concentration with IC_50_ of 20 μg/ml. In BChE inhibition assay, Ir.Cf, Ir.Flv and Ir.EtAc were found most active causing 88.55 ± 0.77, 82.53 ± 0.71 and 72.66 ± 0.78% enzyme inhibition at 1 mg/ml respectively. Median inhibitory concentrations (IC_50_) for these fractions were 70, 40 and 52 μg/ml respectively. Activities of these fractions were comparable to standard drug galanthamine at the same tested concentration. Among different fractions, Ir.Cf exhibited high phenolic (82.60 ± 0.68) and flavonoid (91.45 ± 1.12) contents, which can be linked to its highest enzyme inhibitory activities.

The use of antioxidants may slow the development of Alzheimer’s disease (AD) and diminish neuronal degeneration [35, 36]. Due to deteriorating effects of oxidative stress on human health the interest in naturally occurring antioxidant has been increased [37]. The flavonoids are reported to possess strong BChE inhibitory potential [38]. The age related brain performance can be improved by the use of vegetables and fruits rich in flavonoids which have the capability to neutralize the free radicals [39]. The total phenolic and flavonoid contents can be correlated with the antioxidant potential against free radicals which can lead to cognitive aging and CNS disorders [40]. Phenolic acids have been reported as natural radical scavengers in vegetables, fruits and herbs by several researchers [41]. The antioxidant potential of phenolics is attributed to their redox properties which enables them to give hydrogen or quench the singlet oxygen [42].

The concentrations of the crude plant extracts that inhibited the DPPH, H_2_O_2_ and ABTS radicals by 50% (IC_50_) are presented in the Figure 1. In DPPH free radicals scavenging assay, Ir.Sp, Ir.Flv, Ir.EtAc and Ir.Cf were found most potent with IC_50_ of 0.1, 19, 22 and 28 μg/ml respectively. In H_2_O_2_ scavenging assay, Ir.Flv, Ir.EtAc and Ir.Cf proved IC_50_ of 24, 30 and 34 μg/ml respectively. Likewise, in ABTS free radicals scavenging activity, Ir,Cr, Ir.Aq and Ir.Sp exhibited IC_50_ of 37, 55 and 70 μg/ml respectively. In Linear correlation of total phenolic contents vs DPPH, H_2_O_2_ and ABTS radicals scavenging activities, these exhibited correlation coefficient of R^2^ = 0.641, 0.742 and 0.415 respectively (Figure 5C, D and E). Concentrations of phenolics in different fractions correlated well to antioxidant and anticholinestrase activities. The current investigations suggest that *I. rugosus* is a good source of anticholinesterase and antioxidant compounds. The fractions showing highest activities should be further subjected to isolation of novel, cost effective and safer active compounds useful in the treatment of neurological disorders.

## Conclusion

Based on the current investigations it may be concluded that the Ir.Flv, Ir.Sp and Ir.Cf isolated from *I. rugosus* exhibited anticholinesterase and antioxidant activities comparable to that of standard drugs which can be attributed to their high phenolic contents. The current study suggests further investigations of *I. rugosus* for isolation, purification and characterization of valuable bioactive compounds for the treatment of various neurodegenerative diseases.

## Methods

### Plant collection and extraction

The aerial parts of *I. rugosus* were collected from Dir (U) Khyber Pakhtunkhwa, Pakistan in May 2013. The plant was identified by plant taxonomist at the department of Botany, Shaheed Benazir Bhutto University, Sheringal Dir (U) KP, Pakistan. The plant specimen was deposited at the herbarium of the same University with voucher number (1016AZ). The plant material was cleaned from dust with tap water and then with distilled water till the complete removal of all the extra particles. It was spread on a clean paper and shade dried for 23 days. The plant material was converted into coarse powder with the help of a grinder. The powdered material (11 kg) was soaked in 80% methanol for 15 days and was filtered. The filtrated solution was concentrated under reduced pressure using rotary evaporator (Heidolph Laborota 4000, Schwabach, Germany) at 40°C. Finally, the crude methanolic extract (900 g) was obtained.

### Fractionation

The crude methanolic extract having weight of 900 g was added to separating funnel and mixed with 500 mL of *n*-hexane, chloroform and ethyl acetate and fractionated by successive solvent-solvent extraction method starting from the low polar solvent towards the high polar solvent using method described previously [43]. Finally, Ir.Hex, Ir.Cf, Ir.EtAc and Ir.Aq obtained were 30 g, 45 g, 50 g and 180 g respectively.

### Extraction of crude saponins

Crude saponins from *Isodon rugosus* were extracted by adding 20 g of the plant sample in a conical flask having 100 mL of 20% ethanol. This mixture was heated on water bath at 55°C for 4 h followed by filtration. The residue obtained was extracted again with 200 mL of 20% ethanol and was added to the first collection. Volume of solvent extract was reduced to 40 ml using water bath to obtained greenish color residue. The residue was transferred into a separating funnel and diethyl ether (20 mL) was added to it. After vigorous shaking, the separating funnel was put in a stand to get two layers. The upper organic layer was discarded while the lower aqueous layer obtained was diluted with 60 mL of *n*-butanol. This combined *n-*butanol extract was washed twice with 10 ml of 5% NaCl solution. The solution was evaporated by keeping in water bath to obtain the dried saponins [44].

### Extraction of crude flavonoids

Harborne’s procedure was followed for the isolation of crude flavonoids [45]. Plant sample having weight of 20 g was taken in powdered form and heated in 200 ml of 2 M HCl at 50°C under reflux for 30 minutes. It was allowed to cold and then filtered using whatman No.42 filter paper. The filtrate was treated with equal volume of ethyl acetate. The flavonoids present in the extract were precipitated, which was recovered with the help of weighed filter paper. The weight of flavonoids obtained was 1.5 grams (7.5%).

### Determination of total phenolic contents

The total phenolic contents was determined following the procedure reported previously [46]. Briefly, 1 ml of diluted extract was mixed with 9 ml of distilled water followed by addition of 1 ml of Folin-Ciocalteu’s reagent (FCR) with vigorous shaking. After 5 min, 10 ml of 7% Na_2_CO_3_ solution was added to it and properly mixed. The mixture was diluted to 25 ml with distilled water and shaken properly. After 90 min, the absorbance was measured by using UV spectrophotometer at 750 nm. Gallic acid standard curve was used for the measurement of total phenolic contents. The total phenolics were expressed as milligrams of gallic acid equivalent (mg GAE/g) per gram of dry sample.

### Determination of total flavonoids contents

Total flavonoid contents of *Isodon rugosus* were determined using procedure previously described [47]. Briefly, 0.3 ml of plant sample solution, 0.15 ml NaNO_2_ (0.5 M), 3.4 ml methanol (30%) and 0.15 ml AlCl_3_.6H_2_O (0.3 M) were mixed and added to a 10 ml test tube. After 5 min, 1 ml of NaOH (1.0 M) was added to it. The absorbance of the mixture was measured by using UV spectrophotometer at 506 nm. For the determination of total flavonoids, standard Rutin solution curve (0 to 100 mg/l) was used. The total flavonoid contents were expressed as milligram of rutin equivalent per gram of dry sample (mg RTE/g).

### Chemicals and drugs

Potassium phosphate buffer (pH 8.0), Galantamine from *Lycoris* Sp. (Sigma-Aldrich France), Butyrylthiocholin iodide (Sigma-Aldrich Switzerland), Acetylthiocholine Iodide (Sigma-Aldrich UK), 5,5-dithio-bis-nitrobenzoic acid (DTNB) (Sigma-Aldrich Germany), *Electric eel* acetylcholinesterase (type-VI-S, Sigma-Aldrich USA) and Aquine butyrylcholinesterase (Sigma-Aldrich USA) were used in enzyme inhibition assays. For antioxidant assays, DPPH (Sigma Aldrich CHEMIE GmbH USA), H_2_O_2_ (Riedel-de Haen Germany) and ABTS (Sigma-Aldrich Germany) were used. All solvents used in the assays were of analytical grades purchased from Sigma-Aldrich.

### Anticholinesterase assays

In this method, plant samples were evaluated spectrophotometrically for AChE and BChE inhibition potentials using acetylthiocholine iodide and Butyrylthiocholine iodide as substrates, respectively following Ellman assay [48]. In this method, 205 μl of plant samples (125–1000 μg/ml) were added to the cuvette contained BChE (0.01 U/ml) and 5 μl of AChE (0.03 U/ml). DTNB (5 μl) was added to the mixture and incubated for 15 min in a water bath having temperature of 30°C. To start the reaction added 5 μl substrates to the mixture. Absorbance was measured using double beam Spectrophotometer at wavelength of 412 nm for 4 min.

The reaction between thiocholines and DTNB was indicated by the formation of 5-thio-2-nitrobenzoate anion, which showed by yellow color appearance. White assay was carried out to check the hydrolysis of substrate contained no enzymes and plant samples. The reaction mixture containing without plant sample was taken as control. Percent enzyme activity and percent inhibition were calculated as follows;


(Where V indicates the rate of reaction in the presence of inhibitor and V_max_ is the rate of reaction without inhibitor).

### Antioxidant assays

#### DPPH free radicals scavenging activity

For DPPH free radicals scavenging activity the method of Brand-Williams et al. was followed with some modifications [49]. DPPH (24 mg) was dissolved in 100 ml of methanol to prepare DPPH solution. The stock solutions of plant samples were prepared in methanol having concentrations of 1 mg/ml and then diluted to the concentrations 1000, 500, 250 μg/ml. DPPH and sample solutions were mixed in a ratio of 1:1 and were incubated at 23°C for 30 min. Finally, absorbance was measured at 517 nm using UV spectrophotometer (Thermo electron corporation USA). Ascorbic acid was used as positive control. Percent radical scavenging activity was measured using the following equation;


#### Hydrogen peroxide free radicals scavenging activity

The plant samples were investigated for hydrogen peroxide free radical scavenging potential following the procedure of Ruch *et al.,* [50]. Briefly, 2 mmol solution of hydrogen peroxide was prepared in 50 mmol phosphate buffer having pH of 7.4. Various plant samples (0.1 ml) were taken in test tubes followed by addition of 50 mmol phosphate buffer to make the final volume 0.4 ml. Hydrogen peroxide (0.6 ml) was added to the test tube and kept for 10 min. Absorbance was measured spectrophotometrically at 230 nm against the blank. The hydrogen peroxide scavenging activity was calculated using the following equation;


#### ABTS free radicals scavenging activity

Antioxidant potentials of Ir.Cr, Ir.Hex, Ir.EtAc, Ir.Cf, Ir.Aq, Ir.Flv and Ir.Sp were investigated using the free radicals of 2, 2-azinobis [3-ethylbenzthiazoline]-6-sulfonic acid (ABTS) [51]. ABTS (7 mmol) and potassium persulfate (2.45 mmol) solutions were prepared and thoroughly mixed. For production of free radicals, the solution was kept in the dark for 8 h at room temperature. Prior to use, ABTS solution was diluted with Phosphate buffer (0.01 M), pH 7.4. The absorbance of ABTS solution was adjusted to 0.7 at 745 nm by the addition of 50% methanol. Radical scavenging ability of the fractions was analyzed by mixing 300 μl of each test sample with 3.0 ml of ABTS solution in a cuvette. The absorbance of the solution was measured using a double beam spectrophotometer for 6 min. Ascorbic acid was used as positive control. The experiment was repeated three times and percent ABTS scavenging activity was calculated as follows;


### Statistical analysis

Each experiment was performed in three replicates and values were expressed as mean ± SEM. One way ANOVA followed by multiple comparison Dunnett's test was used for the comparison of positive control with the test groups. The P values less than 0.05 were considered as statistically significant. IC_50_ values were calculated by a linear regression analysis among the percent inhibition against the extract concentrations via Excel program.

### Regression and linear correlation

Regression (y) and linear correlation (R^2^) for phenolic contents and various activities (anticholinesterase and antioxidants) were determined using Microsoft Excel 2007.
